# High risk of adult asthma following severe wheeze in early life

**DOI:** 10.1186/2045-7022-3-S1-O8

**Published:** 2013-05-03

**Authors:** Emma Goksör, Bernt Alm, Mainor Åmark, Linda Ekerljung, Bo Lundbäck, Göran Wennergren

**Affiliations:** 1 Department of Paediatrics, University of Gothenburg, Sweden; 2 Krefting Research Centre, Department of Medicine, University of Gothenburg, Sweden

## Background

We have previously reported on the outcome in childhood and adolescence in children with severe wheeze in early life. The aim of the present follow-up was to report on the asthma prevalence and risk factors for asthma in adult age.

## Methods

We have prospectively studied asthma development in 101 children hospitalized due to wheezing before the age of two. The cohort was re-investigated at age 25-29 years and tested for bronchial hyper-responsiveness and allergic sensitization. The response rate at adult age was 81%. The results were compared to a population based aged matched control group (n=1210) recruited from the West Sweden Asthma Study.

## Results

Current asthma was seen in 37% (30/82) and 50% of these had a moderate to severe asthma. In the control group 10% reported current asthma (OR 5.3, 95% CI 3.2-8.9; p<0.001) and 17% had wheezing during the last 12 months (p<0.001). Current use of asthma medication was reported in 31% of the cohort (of which 66% used inhaled corticosteroids and/or montelukast), compared to 8% in the control group (p<0.001). Current atopy was found in 54%, with 42% reporting doctor-diagnosed rhinitis, 11% current eczema and 16% food allergy. Among the controls rhinitis was reported in 29% (p=0.013) and eczema in 13% (ns). Smoking was reported in 30 % of the cohort, compared to 16% in the control group (p=0.002).

In the cohort, current allergy (OR 9.7, 95% CI 3.0-31.1) and female gender (OR 3.2, 1.1-9.5) increased the risk of adult asthma independently of each other. Females with current allergy had the highest risk of adult asthma, compared to males without allergy (OR 29.4, 5.0-173.3). This is illustrated in a stratified Cox regression analysis where the females with current allergy have the lowest chance of recovery (Hazard Ratio 0.2, 95% CI 0.06-0.5) compared to males without allergy.

## Conclusion

Subjects with severe early wheezing have an increased risk of adult asthma. Females with current allergy had the highest risk of persistent asthma and the lowest chance of recovery.

